# Gerontoxanthone I and Macluraxanthone Induce Mitophagy and Attenuate Ischemia/Reperfusion Injury

**DOI:** 10.3389/fphar.2020.00452

**Published:** 2020-04-15

**Authors:** Qian Xiang, Man Wu, Li Zhang, Wenwei Fu, Jinling Yang, Baojun Zhang, Zhaoqing Zheng, Hong Zhang, Yuanzhi Lao, Hongxi Xu

**Affiliations:** ^1^School of Pharmacy, Shanghai University of Traditional Chinese Medicine, Shanghai, China; ^2^Engineering Research Center of Shanghai Colleges for TCM New Drug Discovery, Shanghai University of Traditional Chinese Medicine, Shanghai, China; ^3^Institute of Cardiovascular Disease of Integrated Traditional Chinese and Western Medicine, Shuguang Hospital affiliated to Shanghai University of Traditional Chinese Medicine, Shanghai, China

**Keywords:** mitophagy, PINK1-Parkin pathway, Gerontoxanthone I, Macluraxanthone, ischemia/reperfusion injury

## Abstract

Mitophagy is a crucial process in controlling mitochondrial biogenesis. Balancing mitophagy and mitochondrial functions is required for maintaining cellular homeostasis. In this study, we found that Gerontoxanthone I (GeX1) and Macluraxanthone (McX), xanthone derivatives isolated from *Garcinia bracteata* C. Y. Wu ex Y. H. Li, induced Parkin puncta accumulation and promoted mitophagy. GeX1 and McX treatment induced the degradation of mitophagy-related proteins such as Tom20 and Tim23. GeX1 and McX directly stabilized PTEN-induced putative kinase 1 (PINK1) on the outer membrane of the mitochondria, and then recruited Parkin to mitochondria. This significantly induced phosphorylation and ubiquitination of Parkin, suggesting that GeX1 and McX mediate mitophagy through the PINK1-Parkin pathway. Transfecting ParkinS65A or pretreated MG132 abolished the induction effects of GeX1 and McX on mitophagy. Furthermore, GeX1 and McX treatment decreased cell death and the level of ROS in an ischemia/reperfusion (IR) injury model in H9c2 cells compared to a control group. Taken together, our data suggested that GeX1 and McX induce PINK1-Parkin–mediated mitophagy and attenuate myocardial IR injury *in vitro*.

## Introduction

Mitochondria are essential organelles for maintaining myocyte functions and survival. Mitochondria provide myocytes with ATP (adenosine triphosphate) *via* oxidative phosphorylation (Youle and Narendra, 2011; Kubli et al., 2015). Mitochondrial proteins are involved in metabolic processes, such as autophagy (especially mitophagy), apoptosis, innate immunity, heart disease, and neurodegenerative disease (Wang et al., 2012; Subramanian et al., 2013; Wu et al., 2019b). Mitochondria are key regulators and important signaling organelles in different tissues (Scialo et al., 2016). However, damaged or dysfunctional mitochondria, which produce less ATP than healthy mitochondria, are harmful to myocytes, as they generate excess reactive oxygen species (ROS) and toxic byproducts. Additionally, damaged or dysfunctional mitochondria are associated with various diseases (Scialo et al., 2016). Therefore, it is important to remove damaged or dysfunctional mitochondria (Wallace, 1999).

Mitophagy can remove damaged or dysfunctional mitochondria, which has been intensively investigated (Dombi et al., 2018). Mitophagy is an autophagic response that specifically targets dysfunctional or damaged mitochondria. Previous studies have indicated that mitophagy maintains a healthy mitochondrial population and mitochondrial quality (Youle and Narendra, 2011). Mitophagy can be triggered by multiple forms of cellular stress, such as starvation, hypoxia, and ROS, which are also associated with some kinds of neurodegeneration and cardiovascular diseases (CVDs) (de Vries and Przedborski, 2013; Dorn and Kitsis, 2015; Ji et al., 2016). For example, Blass et al. have proposed that mitochondrial dysfunction is the early characteristic of Alzheimer’s disease (Blass and Gibson, 1991). Recent studies have illustrated that accumulation of Aβ can impair mitophagy. Impaired mitophagy promotes Aβ and Tau pathologies in Alzheimer’s disease (Kerr et al., 2017). Billia et al. have demonstrated that the protein level of PINK1 is significantly decreased in end-stage human heart failure. In agreement with this, PINK1^-/-^ mice have a greater tendency than wild-type mice to develop pathological cardiac hypertrophy (Billia et al., 2011; Bravo-San Pedro et al., 2017). Current studies have shown that PINK1 and the cytosolic E3 ubiquitin ligase Parkin are the two main regulators of mitophagy in mammalian cells (Hang et al., 2018). PINK1 is a kinase localized to mitochondria. It is maintained at very low levels by being rapidly degraded by proteolysis when it is imported into mitochondria (Youle and Narendra, 2011). When mitochondria are damaged, mitochondrial membrane potential (MMP) decreases. When this occurs, full-length PINK1 accumulates on the outer membrane of mitochondria and recruits Parkin to mitochondria. Upon activation, Parkin then ubiquitinates mitochondrial surface proteins, which leads to recruitment of nuclear dot protein 52 kDa (NDP52), an ubiquitin- and LC3-binding adaptor protein. When NDP52 is recruited to mitochondria, it modulates the process of mitophagy by causing the decrease of mitochondrial mass, finally resulting in elimination of damaged mitochondria (Liu et al., 2012; Park et al., 2012; Pi et al., 2013; Wei et al., 2014).

Cardiovascular disease is the most common cause of morbidity worldwide. Cardiomyocytes heavily rely on ATP produced by mitochondria, so they are more sensitive to mitochondrial dysfunction than many other cell types (Liang et al., 2013; Mortality and Causes of Death, 2015). Myocardial ischemia is caused by initial interruption of blood flow supplying oxygen and nutrients to the heart (Yang et al., 2019). Post-ischemic reperfusion is essential to rescue viable myocardium and to maintain cardiac function (Kambe et al., 2009). Strikingly, the process of reperfusion can induce cardiomyocyte death. This phenomenon is called reperfusion injury (Kambe et al., 2009; Hausenloy and Yellon, 2013). Myocardial ischemia/reperfusion (IR) led to cell death and decreased cardiac output (Mozaffarian et al., 2016). The role of mitophagy in myocardial ischemia/reperfusion injury has drawn extensive recent attention. Mitophagy has a “double effect” in the setting of cardiac IR injury. On one hand, excessive mitophagy can participate in the pathogenesis of cardiac IR injury (Ma et al., 2015; Ishikita et al., 2016); on the other hand, mitophagy is significantly suppressed by IR injury. IR injury can be attenuated *via* inducing mitophagy. PINK1-Parkin-mediated mitophagy is particularly regarded as a novel therapeutic pathway for myocardial IR injury (Zhou et al., 2017; Zhang et al., 2019b). Our study showed that GeX1 and McX have cardioprotective effects by promoting mitophagy in H9c2 cells.

Nowadays, natural plant-derived compounds are widely studied. Gerontoxanthone I (GeX1) and Macluraxanthone (McX), the dimeric xanthones, were extracted from *Garcinia bracteata* C. Y. Wu ex Y. H. Li. In this study, we found that GeX1 and McX directly stabilized PINK1 on the outer membrane of the mitochondria. Then they recruited Parkin to mitochondria and significantly induced Parkin phosphorylation and ubiquitination to promote mitophagy. GeX1 and McX treatment also inhibited cell death in an IR injury model in H9c2 cells.

## Materials and Methods

### Plant Material

GeX1 and McX (with a purity greater than 98%, **Figures S1A, B**) were extracted from the leaves of *Garcinia bracteata* C. Y. Wu ex Y. H. Li, collected from Nanning, Guangxi Province, People’s Republic of China, in October 1981 and authenticated as *G. bracteata* C. Y. W u ex Y. H. Li by Specialist Yu Wan. A voucher specimen (GXMG0020900) was deposited at Guangxi Botanical Gargen of Medicinal Plants.

### Reagents and Antibodies

Carbonyl cyanide m-chlorophenylhydrazone (CCCP) and propidium iodide (PI) were purchased from Sigma. The following antibodies were used for western blot analysis or immunofluorescence: COX IV (4850), NDP52 (60732), and GST (2625) were purchased from Cell Signaling Technology; monoclonal anti-ubiquitin (sc-8017), anti-Tom20 (sc-17764), anti-PINK1 (sc-517353), and anti-Parkin (sc-32282) were purchased from Santa Cruz; anti-LC3B (L7543) was purchased from Sigma; and anti-Tim23 (611222) was purchased from Becton Dickinson and Company. Actin (EM21002) was purchased from HuaBio. GAPDH (ab128915) was purchased from Abcam.

### Cell Culture

H9c2 cells, YFP-Parkin HeLa cells, and SH-SY5Y cells were cultured in Dulbecco’s modified Eagle’s medium (DMEM) (Gibco, C11995500CP) supplemented with 10% (v/v) fetal bovine serum (FBS) (BI, 04-001-1ACS) and 1% penicillin/streptomycin (Genom, GNM15140). All cells were maintained in a humidified incubator with 95% air and 5% CO_2_ at 37°C. For ischemic or hypoxic damage, H9c2 cells were incubated in a hypoxic incubator (1% O_2_, 5% CO_2_, and 94% N_2_) with ischemia-mimetic solution (CaCl_2_, 1.8 mM; NaCl, 135 mM; MgCl_2_, 0.5 mM; KCl, 8 mM; Na^+^-lactate, 20 mM; NaH_2_PO_4_, 0.33 mM; HEPES, 5.0 mM, pH 6.8) (Sun et al., 2016). For reperfusion, H9c2 cells with ischemic or hypoxic damage were rapidly transferred into a normoxic incubator with fresh DMEM.

### Measurement of Cell Survival

Cell viability was determined with propidium iodide. H9c2 cells, YFP-Parkin HeLa cells, and SH-SY5Y cells were plated in 24-well plates and then treated with GeX1 and McX for 24 h. Cells were incubated with 5 μg/mL propidium iodide for 30 min at 37°C and then washed with pre-warmed PBS. The fluorescence of the cells was analyzed by flow cytometry (Becton Dickinson and Company).

### Measurement of Mitochondrial Mass

After treatment with GeX1 and McX for 4 h, SH-SY5Y cells were incubated with 100 nM MitoTracker Green in the dark for 30 min at 37°C. They were then washed with pre-warmed PBS. The fluorescence of the cells was analyzed by flow cytometry.

### Cell Fractionation and Mitochondrial Isolation

SH-SY5Y cells were plated in 10 cm dishes, and they were treated with GeX1 and McX for 0, 4, or 8 h. Using a Cell Mitochondria Isolation Kit (Beyotime), we separated the cytosolic and mitochondrial proteins according to the manufacturer’s instructions. In brief, cells were washed with pre-cooled PBS and lysed with a Cell Mitochondria Isolation buffer on ice. Mitochondria and cytoplasm are separated by grinding followed by centrifugation at 600*g* for 10 min at 4°C. Then the supernatant was further centrifuged at 11,000*g* for 10 min at 4°C. The pellet was collected as the mitochondria-enriched fraction, and it was then further resuspended in mitochondrial lysis buffer. The remaining supernatant was centrifuged 12,000*g* for 10 min at 4°C as cytosolic proteins. Protein concentrations were detected using a Multimode Plate Reader (PerkinElmer). Equal amounts of protein (20 μg) from each fraction were measured by western blotting.

### Immunofluorescence Microscopy

Following treatment with GeX1 and McX for 4 h, YFP-Parkin HeLa cells on coverslips were fixed in 4% paraformaldehyde. Then cells were permeabilized and blocked with 2% goat serum containing 0.5% Triton X-100 and 3% BSA for 1 h at room temperature. The cells were probed with the primary antibodies anti-Parkin and anti-Ub. After three PBS washes, the cells were stained with secondary antibody. All fluorescent images were acquired on a confocal microscope (Olympus).

### Quantitative Real-Time PCR

After treatment of SH-SY5Y cells with GeX1 and McX for 4 h, total RNA was extracted using Trizol reagent (Takara, 9108). RNA was converted to cDNA with the use of a PrimeScript RT reagent kit (TaKara, DRR037A), and relative mRNA levels were measured using SYBR green (TOYOBO, QPK-201). Then, real-time PCR was performed in a StepOnePlus Real-Time PCR System (Applied Biosystems, Life Technologies). The primers for human genes were as follows: for Tim23, forward primer: 5′-CAACATCCTCCAATCGTAAAG-3′, reverse primer: 5′-GGTATGAACCCTCTGTCTCCT-3′; for Tom20, forward primer: 5′-CTGGAACACTGGTGGTGGAAG-3′, reverse primer: 5′-AGGTGAATATGAGAAGGGCGTAG-3′; and for TBP, forward primer: 5′-CCACTCACAGACTCTCACAAC-3′, reverse primer: 5′-CTGCGGTACAATCCCAGAACT-3′.

### Western Blot Analysis

Cells were solubilized in ice-cold whole cell extract buffer (50 mM Tris-HCl, pH 8.0, 4 M urea, and 1% Triton X-100) supplemented with protease inhibitor mixture (Roche Diagnostics, 04693132001). The mixture was clarified by centrifugation at 14,000*g* for 30 min at 4°C. Protein samples were loaded and separated on a 12% sodium dodecyl sulphate–polyacrylamide gel (SDS-PAGE) and transferred to a polyvinylidene fluoride membrane. This was blocked with 5% non-fat milk for 1 h at room temperature in Tris-buffered saline (50 mM Tris-HCl, pH 7.5, 150 mM NaCl) containing 0.2% Tween 20. Blots were probed with the following antibodies: LC3, NDP52, monoclonal anti-ubiquitin, Tom20, PINK1, Parkin, Tim23, Actin, COX IV, or GST. GAPDH, Actin, and COX IV were used as the loading control. Then they were incubated with secondary anti-mouse (KPL, 074-1806) or anti-rabbit antibodies (KPL, 474-1506) for 1.5 h at room temperature. Protein bands were visualized using ECL blotting detection reagents (KPL, 54-61-00).

### Transfection of siRNA or GST-Parkin Plasmid

SH-SY5Y or H9c2 cells were seeded in 12-well plates and transfected with siRNA for 24 or 48 h, using Lipofectamine 2000 (Invitrogen, 11668027) as instructed according to the manufacturer’s specifications. Cells were then treated with GeX1 and McX. PINK1 siRNA (Santa Cruz Biotechnology, sc-44598), Parkin siRNA (rat) and negative control siRNA were purchased from GenePharma. The sequences of PINK1 siRNA were as follows: PINK1 siRNA: 5′-CGCUGUUCCUCGUUAUGAATT-3′; The sequences of Park2 were offered as below: Park2: 5′-CCAACUCCCUGAUUAAAGATTUCUUUAAUCAGGGAGUUGGTT-3′; negative control siRNA (sense), 5′-UUCUCCGAACGUGUCACGUTT-3′; and negative control siRNA (antisense), 5′-ACGUGACACGUUCGGAGAATT-3′. HeLa cells were plated in 12-well plates and transfected with GST-Parkin^WT^ or GST-Parkin^S65A^ plasmid for 24 h, using Lipofectamine 2000 (Invitrogen, 11668027) as instructed according to the manufacturer’s specifications, and then treated with GeX1 and McX.

### Co-Immunoprecipitation

YFP-Parkin HeLa cells were lysed in IP lysis buffer (10 mM Tris-HCl, pH 7.4, 100 mM NaCl, 2.5 mM MgCl_2_, 0.05% Triton-100, with protease inhibitors). This was followed by centrifugation at 500*g* for 10 min at 4°C. The cell supernatants were incubated with 10 μL GFP-Trap (ChromoTek) beads overnight at 4°C with rotation. On the next day, the beads were washed three times with 1 mL of the IP buffer. Then the samples and corresponding total cell lysates were boiled with 2× sample loading buffer and separated by SDS-PAGE for further western blotting. Proteins were transferred onto polyvinylidene difluoride membranes. Membranes were incubated overnight at 4°C with anti-Parkin antibody.

### Statistical Analysis

Each experiment was repeated three times. Date are expressed as means ± SEM. Statistical analyses were performed using the GraphPad Prism software Student’s *t* test.

## Results

### Screening Novel Mitophagy Regulators From *Garcinia* Species

**Figure 1:**
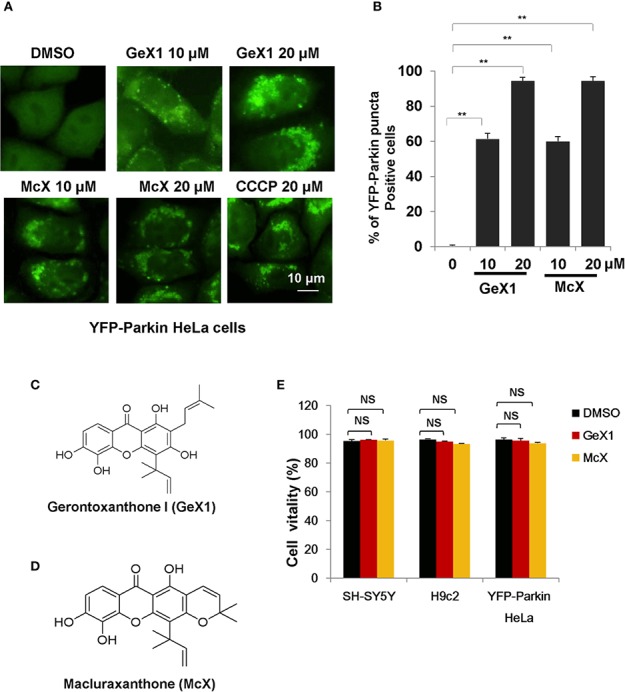
GeX1 and McX induce mitophagy. **(A)** HeLa cells stably expressing YFP-Parkin were treated with xanthone derivatives for 4 h. The distribution of YFP-Parkin was examined by confocal microscopy. Scale bar, 10 μm. **(B)** HeLa cells stably expressing YFP-Parkin were treated with GeX1 or McX for 4 h. Then we performed quantification of YFP-Parkin puncta-positive cells at different drug concentrations (10 or 20 μM), **P < 0.01, treatment with GeX1 and McX compared to DMSO. **(C, D)** The molecular structure of GeX1 and McX. **(E)** Cell proliferation after treatment with GeX1 and McX was measured in SH-SY5Y, H9c2, and YFP-Parkin HeLa cells by propidium iodide exclusion. NS, not significant.

To identify novel mitophagy regulators from *Garcinia* species, we performed a functional screen using a cell-based assay. We treated HeLa cells stably expressing YFP-Parkin with a series of xanthones from *Garcinia* species and observed YFP-Parkin puncta accumulation, which can be used to monitor mitophagy. The most interesting finding was that a group of xanthone compounds isolated from *Garcinia bracteata* C. Y. Wu ex Y. H. Li, represented by GeX1 and McX (Zhang et al., 2019a), intensively promoted the accumulation of Parkin puncta. We found that GeX1 and McX significantly induced a dose-dependent increase of Parkin puncta accumulation, suggesting that these compounds can regulate mitophagy (**Figures 1A–D**). The toxicity of these two compounds in YFP-Parkin HeLa, SH-SY5Y, and H9c2 cells were tested. Propidium iodide exclusion assay results showed that GeX1 and McX have no obvious toxicity to cells at 20 μM for 24 h treatment (**Figure 1E**).

### GeX1 and McX Promote Parkin-Dependent Mitochondrial Degradation

**Figure 2:**
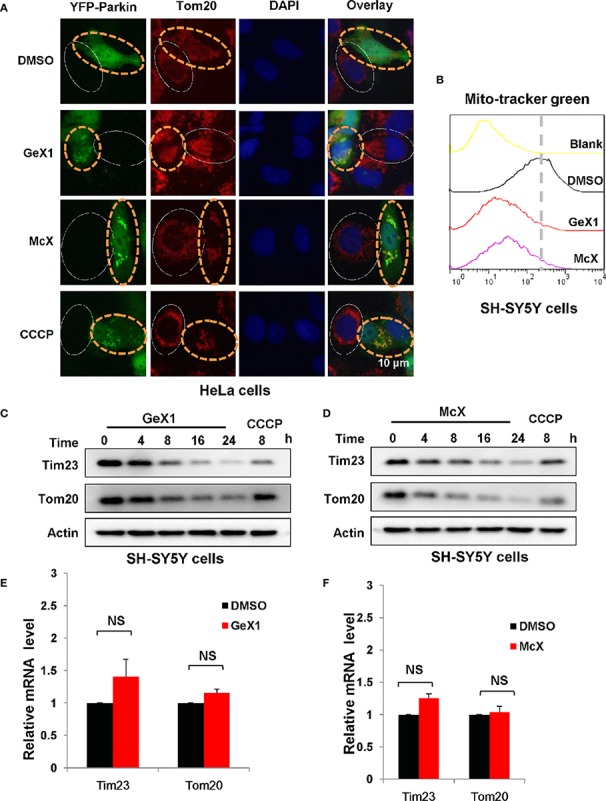
GeX1 and McX promote the removal of mitochondria. **(A)** HeLa cells were transfected with eGFP-Parkin for 24 h and then treated with GeX1 or McX (20 μM) for 6 h. The cells were stained with Tom20 (red). Representative fluorescence microscopy images were shown. The cells marked with the white solid ovals expressed no or low levels of Parkin and still had mitochondria, whereas cells marked with yellow dotted ovals expressed a high level of Parkin and had no mitochondria. **(B)** SH-SY5Y cells were treated with GeX1 or McX (20 μM) for 4 h. The cells were stained with MitoTracker Green and subjected to flow cytometric analysis. **(C, D)** SH-SY5Y cells were treated with GeX1 or McX (20 μM) at the indicated times and analyzed by western blot. Actin was used as a loading control. **(E, F)** SH-SY5Y cells were treated with GeX1 or McX (20 μM) for 4 h and analyzed by RT-PCR. Data for Tim23 or Tom20 mRNA levels compared with actin are shown (mean ± SEM of 3 independent experiments). NS, not significant.

Previous studies have revealed that Parkin plays an important role in maintaining mitochondrial integrity by degradation of damaged mitochondria during mitophagy (Choo et al., 2012), so we investigated whether GeX1 and McX promote Parkin-dependent mitochondrial degradation. We transfected exogenous Parkin into HeLa cells and performed immunostaining assays. As shown in **Figure 2A**, GeX1 and McX had markedly reduced red fluorescence intensity of Tom20 (an outer mitochondrial membrane protein) in HeLa cells transfected with YFP-Parkin, which also inhibited MitoTracker Green staining in SH-SY5Y cells (**Figure 2B**). However, Tom20 (Red fluorescence) did not decrease in cells without Parkin (No green fluorescence cells) (**Figure 2A**). These results suggested that these two compounds increased the removal of mitochondria, and this required the participation of Parkin. We also observed that the protein levels of Tom20 and Tim23 (an inner mitochondrial membrane protein) were degraded in a time-dependent manner in the presence of GeX1 and McX in SH-SY5Y cells. This suggested that GeX1 and McX promoted Parkin-dependent mitochondrial degradation (**Figures 2C, D**). Furthermore, the Q-PCR results suggested that GeX1 and McX treatment had no effect on the mRNA levels of Tim23 and Tom20 (**Figures 2E, F**), suggesting that GeX1 and McX promoted the degradation of these proteins.

### GeX1 and McX Trigger Parkin Phosphorylation and Ubiquitination

It has been well established that phosphorylation of Parkin at Ser65 mediates global polyubiquitination of the outer mitochondrial membrane proteins and regulates proper execution of mitophagy (Kondapalli et al., 2012; Lazarou et al., 2015). We examined the translocation of Parkin after GeX1 and McX treatment. We observed marked colocalization of Parkin and Tom20 (red) in YFP-Parkin HeLa cells treated with GeX1 or McX (**Figure S2**), suggesting that these compounds could promote Parkin transfer to mitochondria, consistent with previous studies.

**Figure 3:**
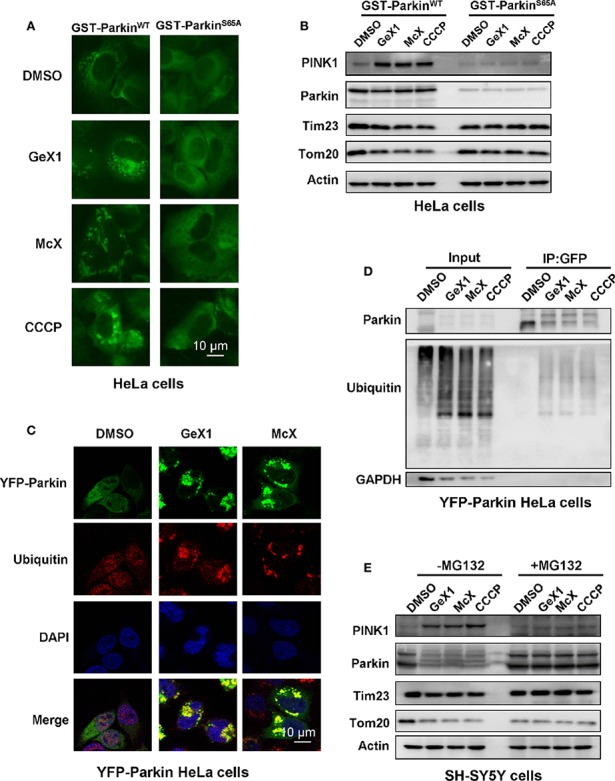
GeX1 and McX trigger Parkin phosphorylation and ubiquitination. **(A)** HeLa cells were transfected with GST-Parkin^WT^ or GST-Parkin^S65A^ mutant for 24 h and then treated with GeX1 or McX (20 μM) for 4 h. **(B)** The GST-Parkin^S65A^ mutant can reverse the effects of GeX1 and McX (20 μM) on the induction of protein upregulation or downregulation. **(C)** YFP-Parkin HeLa cells were treated with GeX1 or McX (20 μM) for 4 h, and were then stained with anti-ubiquitin, followed by confocal microscopy. Scale bars, 10 µm. **(D)** YFP-Parkin HeLa cells were treated with GeX1 or McX (20 μM) for 4 h and analyzed using immunoprecipitation followed by western blotting. **(E)** MG132 pre-treatment for 2 h prevented GeX1 and McX (20 μM) from inducing protein increases or decreases.

When PINK1 stably accumulates on the outer mitochondrial membrane, it will active and recruit Parkin to mitochondria. Several studies reported that PINK1 phosphorylates Parkin serine 65 (Ser^65^) leading its activation (Koyano et al., 2014; Bingol and Sheng, 2016; Kazlauskaite et al., 2015).We wondered whether GeX1 and McX affect the phosphorylation of Parkin at Ser^65^. To address this, we transfected GST-Parkin^WT^ or GST-Parkin^S65A^ mutant plasmids into HeLa cells to detect the translocation of Parkin and the expression of mitochondrial related proteins. Strikingly, immunostaining results revealed that GeX1 and McX treatment induced Parkin puncta formation in GST-Parkin^WT^ transfected cells. In contrast, the puncta were hardly found in cells transfected with mutant GST-Parkin^S65A^ (**Figure 3A**), which illustrated that GeX1 and McX-induced mitophagy is related to Parkin Ser^65^ phosphorylation. The Western blot results showed that GST-Parkin^S65A^ mutant reversed the degradation of Parkin and Tim23 after GeX1 or McX treatment (**Figure 3B**), suggesting GeX1 and McX could phosphorylate Parkin at Ser^65^.

Parkin is an RBR-type E3 ligase that normally localizes in the cytosol as an autoinhibited form, but it translocates to damaged mitochondria to ubiquitinate mitochondrial proteins during mitophagy (Yamano et al., 2016). Therefore, we tested whether GeX1 and McX triggered ubiquitination of Parkin and promoted mitochondria clearance. First, we performed confocal immunofluorescence microscopy analysis testing ubiquitin (red) distribution in YFP-Parkin HeLa cells. Under normal conditions, ubiquitin was spread throughout the cell. When YFP-Parkin HeLa cells were treated with GeX1 or McX, ubiquitin was colocalized with Parkin puncta (**Figure 3C**). Second, we treated cells with GeX1 or McX and found that this increased the expression of ubiquitin. Co-immunoprecipitation assay results further suggested that GeX1 and McX could increase the ubiquitination of Parkin (**Figure 3D**). Finally, we observed that SH-SY5Y cells treated with GeX1 or McX had no effect on protein degradation in the presence of MG132 (a proteasome inhibitor) (**Figure 3E**), suggesting that GeX1 and McX increased Parkin ubiquitination. In summary, GeX1 and McX induce mitophagy *via* inducing the phosphorylation and ubiquitination of Parkin.

### GeX1 and McX Induce Mitophagy Depending on the Stabilization of PINK1

**Figure 4:**
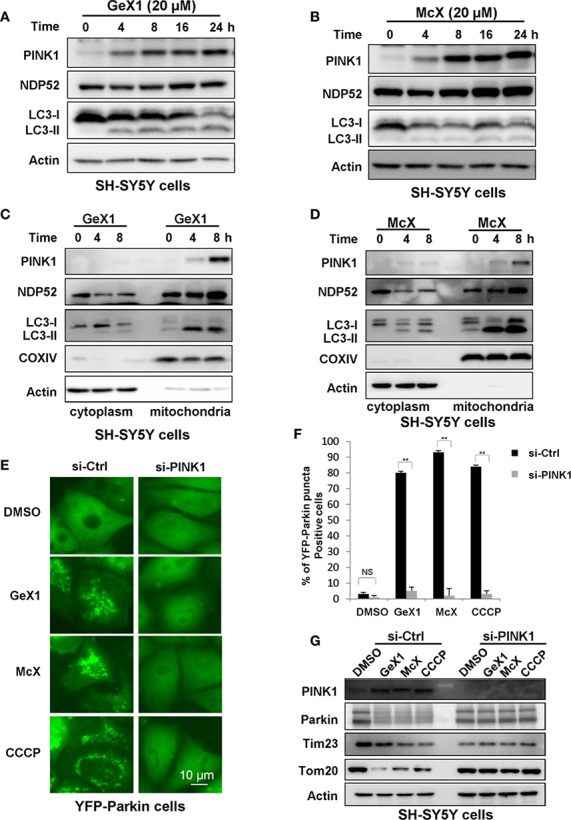
GeX1 and McX induce mitophagy in a manner dependent on PINK1. **(A, B)** The expression of mitophagy-related proteins follows GeX1 and McX treatment of SH-SY5Y cells. **(C, D)** A redistribution of mitophagy-related proteins followed GeX1 and McX (20 μM) treatment of SH-SY5Y cells. **(E, F)** YFP-Parkin HeLa cells were transfected with siPINK1 for 24 h and then treated with GeX1 and McX (20 μM). The distribution of YFP-Parkin was examined by confocal microscopy. Scale bars, 10 µm. NS, not significant. **P < 0.01, PINK1 knockdown YFP-Parkin HeLa cells compared to YFP-Parkin HeLa cells. **(G)**. YFP-Parkin HeLa cells were transfected with siPINK1 for 24 h, and then treated with GeX1 and McX (20 μM). Then related proteins were analyzed by Western blot.

The PINK1-Parkin pathway is a canonical mechanism to mediate mitophagy in most mammalian cells, with PINK1 acting upstream of Parkin (Wei et al., 2014; Qu et al., 2015; Georgakopoulos et al., 2017). PINK1 is an extremely unstable mitochondrial protein. It is constitutively proteolyzed by the mitochondrial rhomboid protease, presenilin-associated rhomboid-like (PARL), at the healthy mitochondria (Muqit et al., 2006; Takatori et al., 2008). However, once mitochondria are damaged, PINK1 degradation is blocked, and PINK1 will accumulate on the outer mitochondrial membrane (OMM), activating Parkin (Kane et al., 2014). We next verified whether the GeX1- and McX-mediated promotion of mitophagy depended on PINK1. Western blot results suggested that GeX1 and McX treatment increased the accumulation of PINK1 in SH-SY5Y cells (**Figures 4A, B**). There are many unique adaptor proteins termed autophagy receptors which play important roles during the mitophagy process (Padman et al., 2019). Some studies have reported that PINK1 recruits NDP52 (the primary receptor for PINK1-Parkin-mediated mitophagy) to mitochondria, where it binds to LC3, completing the mitophagy process (Lazarou et al., 2015). We therefore analyzed the redistribution of NDP52 and LC3. GeX1 and McX treatment upregulated the expression of NDP52 and LC3 II in a dose- and time-dependent manner in SH-SY5Y cells (**Figures 4A, B** and **S3A, B**). To confirm that PINK1, NDP52, and LC3 II translocated to mitochondria followed GeX1 and McX treatment, we prepared mitochondrial and cytoplasmic fractions. GeX1 and McX promoted the accumulation of PINK1, NDP52, and LC3 II on mitochondria in SH-SY5Y cells (**Figures 4C, D**), consistent with previous studies. Furthermore, we employed small interfering RNA (siRNA) to knock down PINK1 in SH-SY5Y cells and investigated the effects of GeX1 and McX on mitophagy. In contrast to si-Ctrl, treatment with GeX1 and McX had no effect on Parkin puncta formation in YFP-Parkin HeLa cells knocked down for PINK1 (**Figures 4E, F**). Western blot results suggested that GeX1 and McX had no effect on the accumulation of PINK1 or on the degradation of mitochondrial-related proteins (Tom20 and Tim23) after PINK1 was knocked down (**Figure 4G**). These findings suggested that GeX1- and McX-induced mitophagy depends on the stabilization of PINK1.

### GeX1 and McX Attenuate IR Injury in H9c2 Cells

**Figure 5:**
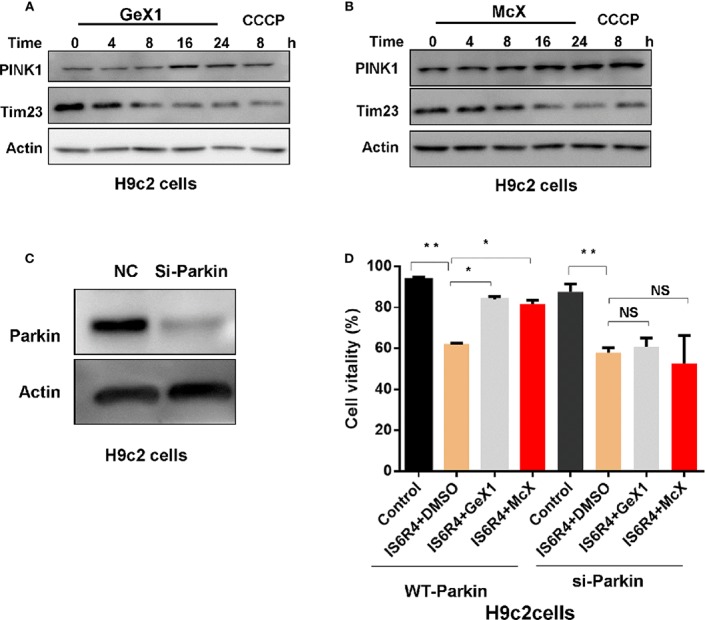
GeX1 and McX attenuate IR injury in H9c2 cells. **(A, B)** H9c2 cells were treated with GeX1 and McX for 4, 8, 16, and 24 h, then analysed the expression of mitophagy related proteins. **(C)** H9c2 cells were transfected with siRNA for 48 h, then measured the expression of Parkin using western blot. **(D)** H9c2 cells or Parkin knockout H9c2 cells were subjected to 6 h of ischemia followed by 4 h of reoxygenation. GeX1 and McX were administered at the onset of reoxygenation. Cell proliferation was detected by propidium iodide exclusion. NS, not significant. *P < 0.05,**P < 0.01, GeX1 and McX treatment cells compared to no treatment.

Cardiomyocytes are sensitive to IR injury caused by insufficient oxygen supply. IR injury can lead to mitochondria damaged, which further harm to cardiomyocytes. Therefore, it is necessary to remove damaged mitochondria *via* mitophagy for the maintenance of cardiomyocyte survival (Kubli and Gustafsson, 2012; Moyzis et al., 2015; Li et al., 2019). Our precious results illustrated that GeX1 and McX could induce mitophagy in SH-SY5Y cells, thus we wondered whether GeX1 and McX can rescue the cell death in IR injury model *via* inducing mitophagy. First, we analysed the effect of GeX1 and McX on mitophagy in H9c2 cells. As shown in **Figures 5A, B**, GeX1 and McX treatment upregulated the expression of PINK1 and degraded Tim23 in time dependent way. In order to investigate the effects of GeX1 or McX on ischemia/reperfusion injury, we established an IR injury model *in vitro*, GeX1 and McX were added at the beginning of reoxygenation. As shown in **Figure 5D**, cell viability increased with the treatment of GeX1 or McX at the beginning of reperfusion. We then evaluated the roles of GeX1 and McX in IR injury after Parkin knockdown (Parkin-KD) cells (**Figure 5C**). However, GeX1 and McX could not rescue the cell death after IR injury in Parkin-KD H9c2 cells, indicating that GeX1 and McX could protect H9c2 cells from IR injury by inducing mitophagy.

## Discussion

Natural compounds are important resources for drug discovery. Xanthones and polycyclic polyprenylated acylphloroglucinols (PPAPs) are the major chemicals from *Garcinia bracteata* C. Y. Wu ex Y. H. Li plants. These types of chemicals exhibit multiple bioactivities. For example, 7-methoxy-4′,6-dihydroxy-8-isobutyryl-flavone has anti-tobacco mosaic virus (TMV) activity (Li et al., 2015); 1,4,5,6-tetrahydroxyxanthone displays anti-cancer activity (Niu et al., 2012); and Scortechinone B, isolated from *Garcinia bracteata* C. Y. Wu ex Y. H. Li, shows antibacterial activity toward methicillin-resistant *Staphylococcus aureus* (Wu et al., 2019a). In this study, we used YFP-Parkin HeLa cells to screen mitophagy regulators and found that GeX1 and McX markedly promoted Parkin puncta accumulation. We then determined that GeX1 and McX induced mitophagy through the PINK1-Parkin pathway, which also attenuated IR injury in H9c2 cells.

Mitochondria are essential double-membrane intracellular organelles that are necessary for cell integrity, function, and survival. Mitochondria can produce ATP *via* oxidative phosphorylation and regulate cellular energy metabolism (Wu et al., 2019a). Mitophagy is responsible for removing damaged, dysfunctional, and redundant mitochondria to maintain mitochondrial biogenesis and functions. The PINK1-Parkin pathway is well known to play a central role in regulating mitophagy. Under physiological conditions, PINK1 is transported to the mitochondrial membrane and then is cleaved and degraded by PARL protein (Yamano and Youle, 2013). However, when mitochondria are damaged, PINK1 accumulates on the OMM and recruits Parkin to ubiquitinate mitochondrial proteins. Subsequently, these proteins are engulfed into autophagosomes, and mitophagy is induced. To determine the effects of GeX1 and McX on mitophagy, we performed western blot to detect the protein expression levels of PINK1, Tom20, and Tim23. We observed that GeX1 and McX stabilized PINK1 on the mitochondrial outer membrane and promoted the degradation of Tom20 and Tim23. Furthermore, GeX1 and McX significantly triggered the phosphorylation and ubiquitination of Parkin. These results suggested that GeX1 and McX induced the PINK1-Parkin dependent pathway. The autophagy receptor NDP52 recognizes ubiquitinated mitochondria. As shown in previous studies, NDP52 binds to autophagosomes *via* ubiquitin and LC3 binding domains during the process of mitophagy (Wong and Holzbaur, 2014; Lazarou et al., 2015). Consistent with these studies, we observed that the expression of NDP52 was increased in the mitochondria of GeX1- and McX-treated cells.

Dysfunctional mitochondria and impaired mitophagy are known to participate in the process of cancer, neurodegenerative diseases, aging, and CVDs (Yoo and Jung, 2018; Yang et al., 2019). Previous studies demonstrated that transient myocardial ischemia leads to cardiac dysfunction, and the stage of reperfusion is particularly injurious to mitochondria (Lesnefsky et al., 2001; Heusch, 2015). The causes of damage mainly include (1) production of excessive ROS, (2) injuries to the mitochondrial respiratory chain, (3) release of Ca^2+^ and cytochrome c to the cytosol, and (4) loss of mitochondrial membrane potential (Shenouda et al., 2011; Ong and Gustafsson, 2012; Ma et al., 2017; Bian et al., 2018). Healthy mitochondria are critical for various biological processes, while damaged mitochondria are cytotoxic to tissues and organs. It is necessary to clear the damaged mitochondria. PINK1-Parkin–mediated mitophagy may be a potential therapeutic target for protecting against myocardial IR injury. IR injury process can lead to insufficient mitophagy. For instance, a recent study found that mitophagy deficiency could induce cardiomyocyte injury and dysfunction at physiological level, and the adequate mitophagy is necessary for cardiomyocyte survival and function followed IR injury (Li et al., 2019). Ling et al. agreed that Polydatin could prevent myocardial IR injury *via* promoting autophagic flux to remove damaged mitochondria to reduce ROS in mice (Ling et al., 2016). However, other groups demonstrated that PINK1/Parkin-mediated mitophagy played advanced effects during IR injury. Zha et al. showed that the advanced glycation end products treatment increased the activation of PINK1/Parkin–mediated mitophagy in the process of cardiomyocyte senescence, thus inhibition of mitophagy might be a promising approach to block the senescent state in cardiomyocytes (Zha et al., 2017). In addition, Woodall et al. suggested that Parkin is dispensable for mitochondrial quality control in a mtDNA mutation mice model of cardiac aging (Woodall et al., 2019). Zhou et al. reported that melatonin may activate AMPKα to suppress mitophagy and protect cardiac microvasculature against IR injury (Zhou et al., 2017). Taken together, we speculate that these findings may vary with the different cell types or animal models, and the relationships between mitophagy and cardioprotection still need further investigation. Our study found that the compounds GeX1 and McX decreased the cell death. GeX1 and McX had a protective effect against myocardial IR injury in H9c2 cells. The mechanism of protective effect of GeX1 and McX against myocardial IR injury needs to be further investigated in an animal model.

Taken together, our findings indicated that GeX1 and McX induce PINK1-Parkin dependent mitophagy. The primary autophagic receptor NDP52 was increased, leading to the degradation of damaged mitochondria by mitophagy. GeX1 and McX attenuated ischemia and reperfusion injury in H9c2 cells by reducing ROS levels. These ﬁndings provide new insights into the molecular mechanisms of natural compounds and explore the function of mitophagy in protection against myocardial IR injury.

## Data Availability Statement

The raw data supporting the conclusions of this article will be made available by the authors, without undue reservation, to any qualified researcher.

## Author Contributions

QX and MW carried out main experimental work and prepared the manuscript. LZ helped with data analysis. LZ, ZZ, and HZ contributed to manuscript revision. JY, BZ, and WF proved compounds. YL and HX designed and supervised this study.

## Funding

This work was supported by National Natural Science Foundation of China (nos. 81973438, 81773951 and 81603344), National Major Scientific and Technological Special Project for “Significant New Drugs Development” in 2018 (No. 2019ZX09301140), and the Three-year development plan project for Traditional Chinese Medicine (ZY (2018–2020)-CCCX-2001-02) to HX.

## Conflict of Interest

The authors declare that the research was conducted in the absence of any commercial or financial relationships that could be construed as a potential conflict of interest.
