# Supplementary material for: Gerontoxanthone I and Macluraxanthone Induce Mitophagy and Attenuate Ischemia/Reperfusion Injury
Source: Front Pharmacol. 2020 Apr 15;11:452. doi: 10.3389/fphar.2020.00452 (PMC7175665; doi:10.3389/fphar.2020.00452)
Supplement: Supplementary file 1 [file DataSheet_1.pdf]

Supplementary Material

1 Supplementary Method

Materials

The purity of GeX1 and McX was more than 98% based on Ultra performance liquid chromatography (UPLC) analysis. UPLC was performed using a Waters Acquity UPLC I class system (Waters, Milford, MA, USA), equipped with a binary solvent delivery system, an auto sampler and a photodiode array detection system. Chromatography was performed on a Waters ACQUITY BEH C_{18} column (2.1 mm × 100 mm, 1.7 μm, Waters). The mobile phase consisted of (A) 0.1% formic acid in water and (B) acetonitrile. The UPLC eluting conditions were as follows: 60–70% (0–2 min) B, 70–90% (2–7 min) B and 90–100% (7–12 min) B. The flow rate was maintained at 0.3 mL•min^{-1}.The column and auto sampler were maintained at 40 ℃ and 10 ℃ respectively.

Immunofluorescence Microscopy

Following treatment with GeX1 and McX for 4 h, YFP-Parkin HeLa cells on coverslips were fixed in 4% paraformaldehyde. Then cells were permeabilized and blocked with 2% goat serum containing 0.5% Triton X-100 and 3% BSA for 1 h at room temperature. The cells were probed with the primary antibodies anti-Tom20. After three PBS washes, the cells were stained with secondary antibody. All fluorescent images were acquired on a microscope (Olympus).

Western blot analysis

Cells were solubilized in ice-cold whole cell extract buffer (50 mM Tris-HCl, pH 8.0, 4 M urea, and 1% Triton X-100) supplemented with protease inhibitor mixture (Roche Diagnostics, 04693132001). The mixture was clarified by centrifugation at 14,000 g for 30 minutes at 4 ℃. Protein samples were loaded and separated on a 12% sodium dodecyl sulphate–polyacrylamide gel (SDS-PAGE) and transferred to a polyvinylidene fluoride membrane. This was blocked with 5% non-fat milk for 1 h at room temperature in Tris-buffered saline (50 mM Tris-HCl, pH 7.5, 150 mM NaCl) containing 0.2% Tween 20. Blots were probed with the following antibodies: LC3, NDP52, PINK1, Actin. Then they were incubated with secondary anti-mouse (KPL, 074-1806) or anti-rabbit antibodies (KPL, 474-1506) for 1.5 h at room temperature. Protein bands were visualized using ECL blotting detection reagents (KPL, 54-61-00).

2 Supplementary Figures

Supplementary Figure 1

A GeX1

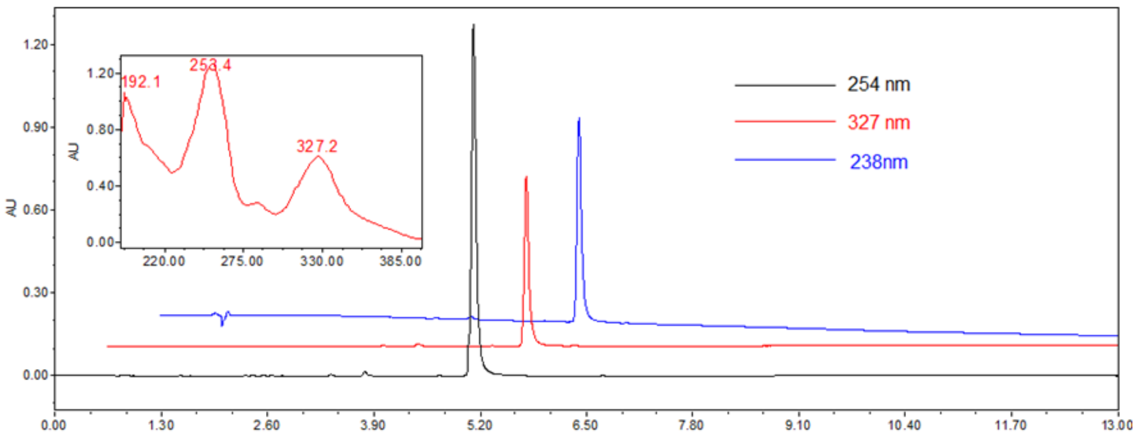

Retention time (min)	Detection wavelength	Purity
5.114	238nm	98.89%
5.114	254nm	98.74%
5.114	327nm	98.71%

B McX

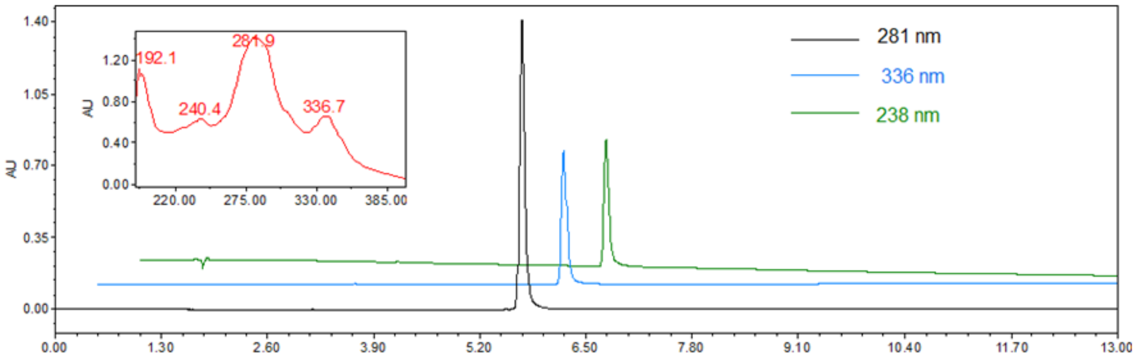

Retention time (min)	Detection wavelength	Purity
5.707	238nm	99.56%
5.707	281nm	99.35%
5.707	336nm	99.81%

Supplementary Figure 1. UPLC chromatogram of GeX1 and McX. Detection wavelength: A. 238, 254, 327 nm (GeX1); B. 238, 281 and 336 nm (McX).

Supplementary Figure 2

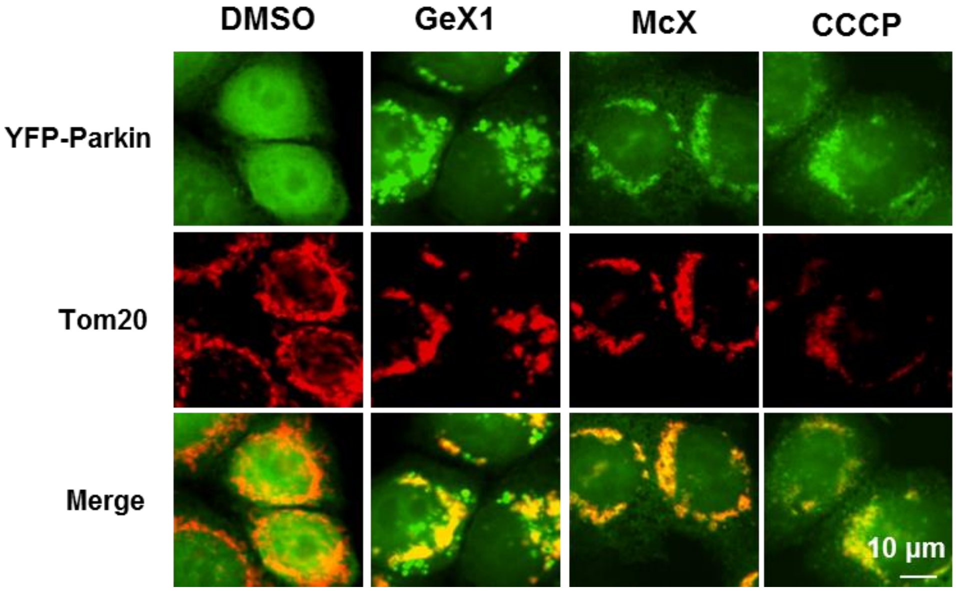

Supplementary Figure 2. GeX1 and McX can promote Parkin translocation to mitochondria. YFP-Parkin HeLa cells were treated with GeX1 and McX for 4 h and then fixed for immunostaining with anti-Tom20 (red). Representative fluorescence microscopy images are shown. Scale bar, 10 µm..

Supplementary Figure 3

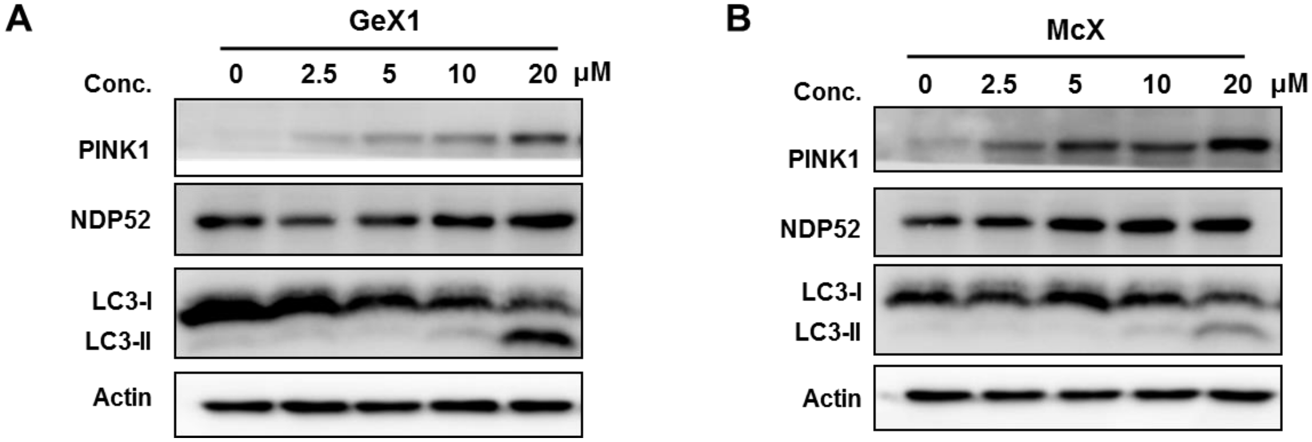

Supplementary Figure 3. A-B. GeX1 and McX promoted mitophagy, as shown by dose dependent increases in the levels of PINK1, NDP52, and LC3-II in SH-SY5Y cells by western blot analysis.
